# Supplementary material for: Systematic genetic characterization of the human PKR kinase domain highlights its functional malleability to escape a viral substrate mimic
Source: bioRxiv. 2024 Jun 5:2024.05.29.596416. Preprint. [Version 2] doi: 10.1101/2024.05.29.596416 (PMC11188142; doi:10.1101/2024.05.29.596416)
Supplement: Supplementary file 1 [file NIHPP2024.05.29.596416V2-supplement-1.pdf]

bioRxiv preprint doi: https://doi.org/10.1101/2024.05.29.596416; this version posted June 5, 2024. The copyright holder for this preprint (which was not certified by peer review) is the author/funder. This article is a US Government work. It is not subject to copyright under 17 USC 105 and is also made available for use under a CC0 license.

SUPPLEMENTAL FIGURES

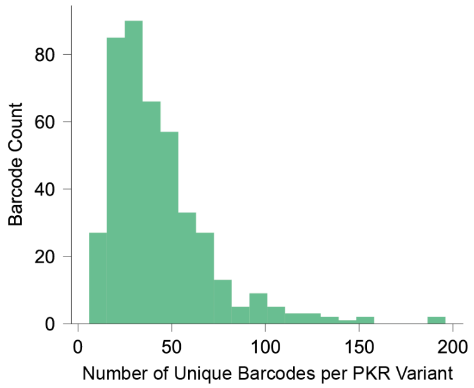

Supplemental Figure 1. Count of unique barcode sequences per PKR variant. Histogram depicts the number of unique barcodes linked to each PKR variant, with a mean of 42.83.

bioRxiv preprint doi: https://doi.org/10.1101/2024.05.29.596416; this version posted June 5, 2024. The copyright holder for this preprint (which was not certified by peer review) is the author/funder. This article is a US Government work. It is not subject to copyright under 17 USC 105 and is also made available for use under a CC0 license.

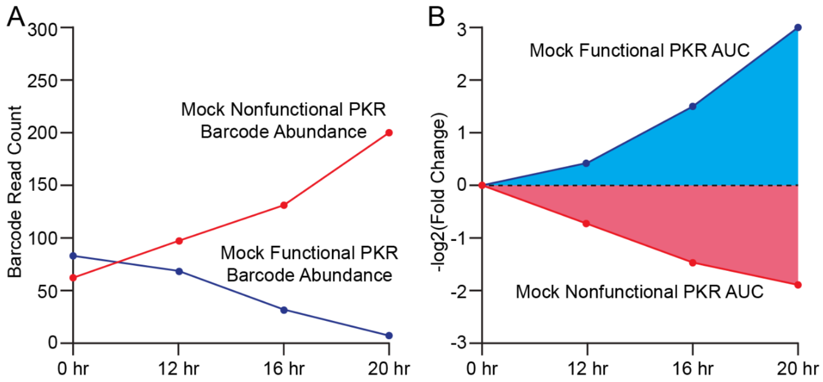

Supplemental Figure 2. Calculation of PKR functional scores from yeast growth assay. (A) Line plot of mock barcode read count data over time for cells expressing nonfunctional (red) or functional (blue) PKR across four sampled timepoints. As PKR activity is toxic to yeast, the number of cells in the pool expressing the functional PKR will decrease over time, and thus the associated barcode read count will also decrease, while the read count for the nonfunctional PKR will increase. (B) Line plot depicting the fold changes from timepoint 0 of barcode abundance derived from Panel A, with a -log2 transformation to assign positive values to functional PKR and negative values to nonfunctional PKR, from which the area under the curve (AUC) is calculated to produce a PKR functional score.

bioRxiv preprint doi: https://doi.org/10.1101/2024.05.29.596416; this version posted June 5, 2024. The copyright holder for this preprint (which was not certified by peer review) is the author/funder. This article is a US Government work. It is not subject to copyright under 17 USC 105 and is also made available for use under a CC0 license.

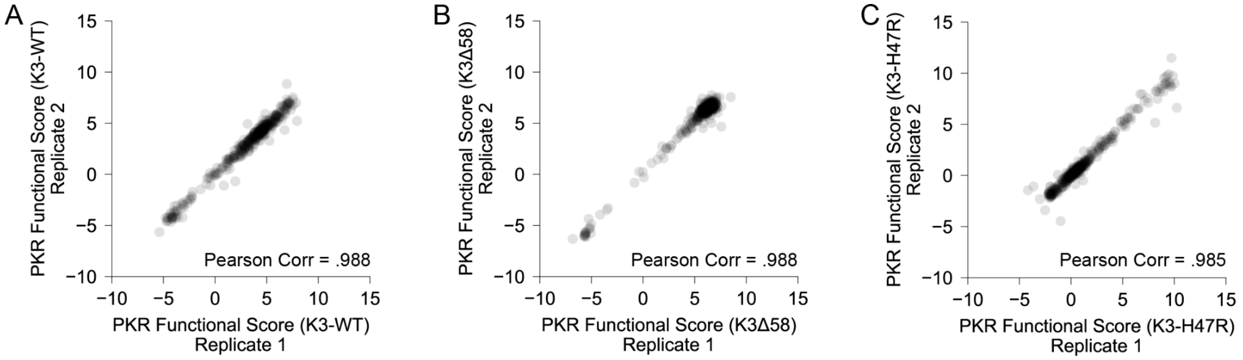

Supplemental Figure 3. Replication of PKR variants paired with K3 alleles. (A) Scatterplot of PKR functional scores for variants paired with K3-WT for two biological replicate experiments. Functional scores were calculated across four timepoints as the area under the curve (see Materials and Methods). Thus, variants with increased evasion of K3 would have higher PKR functional scores, while those with increased susceptibility to K3 or loss of eIF2α kinase activity would have lower scores.

bioRxiv preprint doi: https://doi.org/10.1101/2024.05.29.596416; this version posted June 5, 2024. The copyright holder for this preprint (which was not certified by peer review) is the author/funder. This article is a US Government work. It is not subject to copyright under 17 USC 105 and is also made available for use under a CC0 license.

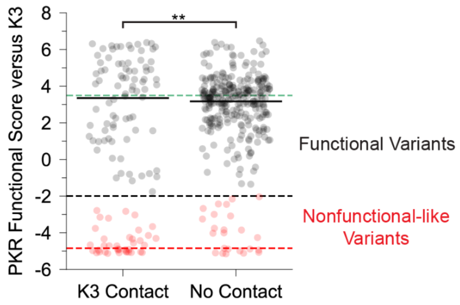

Supplemental Figure 4. Bimodal distribution of PKR functional scores at K3 contact sites. Stripplot of PKR functional scores versus K3 partitioned by predicted contact with K3. Black dashed line denotes the threshold at which variants were separated as nonfunctional-like or functional. Green and red dashed lines represent mean scores for WT PKR and nonsense variants. (** $p < 0.01$, two-sample t-test, comparing just the variants classified as functional in the two classes.)

bioRxiv preprint doi: https://doi.org/10.1101/2024.05.29.596416; this version posted June 5, 2024. The copyright holder for this preprint (which was not certified by peer review) is the author/funder. This article is a US Government work. It is not subject to copyright under 17 USC 105 and is also made available for use under a CC0 license.

```
                                                                   #
                0         10        20        30        40        50
Human eIF2α     MPGLSCRFYQHKFPEVEDVVMVNVRSIAEMGAYVSLLEYNNIEGMILLSELSR--RRIRS
                     1         10        20        30        40        50
Vaccinia K3     -----MLAFCYSLPNAGDVIKGRVYE-KDYALYIYLFDYPHFEAILAESVKMHMDRYVEY
                         : :.:*:. **:  .* .  : . *: *::* ::*.::  *   .  * :

                  60        70        80        90        100       110
Human eIF2α     INKLIRIGRNECVVVIRVDKEKGYIDLSKRRVSPEEAIKCEDKFTKSKTVYSILRHVAEV
                55     60        70        80
Vaccinia K3     RDKL--VGKTVKVKVIRVDYTKGYIDVNYKRMCRHQ------------------------
                 :**  :*..  * *****  *****:. .*:.  :
```

Supplemental Figure 5. Sequence similarity between human eIF2α and vaccinia K3. Alignment of human eIF2α (RefSeq Accession NP_004085.1, residues 0-117) to vaccinia K3 (RefSeq Accession YP_232916.1). The Ser51 site of phosphorylation in eIF2α is indicated by #. Sequences were aligned using Muscle 3.8 and displayed in Clustal format, * = fully conserved, : = strong group conservation, . = weak group conservation. Sites 41-58 of K3 correspond to the rigid helix insert, which is proximal to PKR's ATP-binding site in the Alphafold2-predicted complex, while sites 72-83 are proximal to PKR's helix αG.

bioRxiv preprint doi: https://doi.org/10.1101/2024.05.29.596416; this version posted June 5, 2024. The copyright holder for this preprint (which was not certified by peer review) is the author/funder. This article is a US Government work. It is not subject to copyright under 17 USC 105 and is also made available for use under a CC0 license.

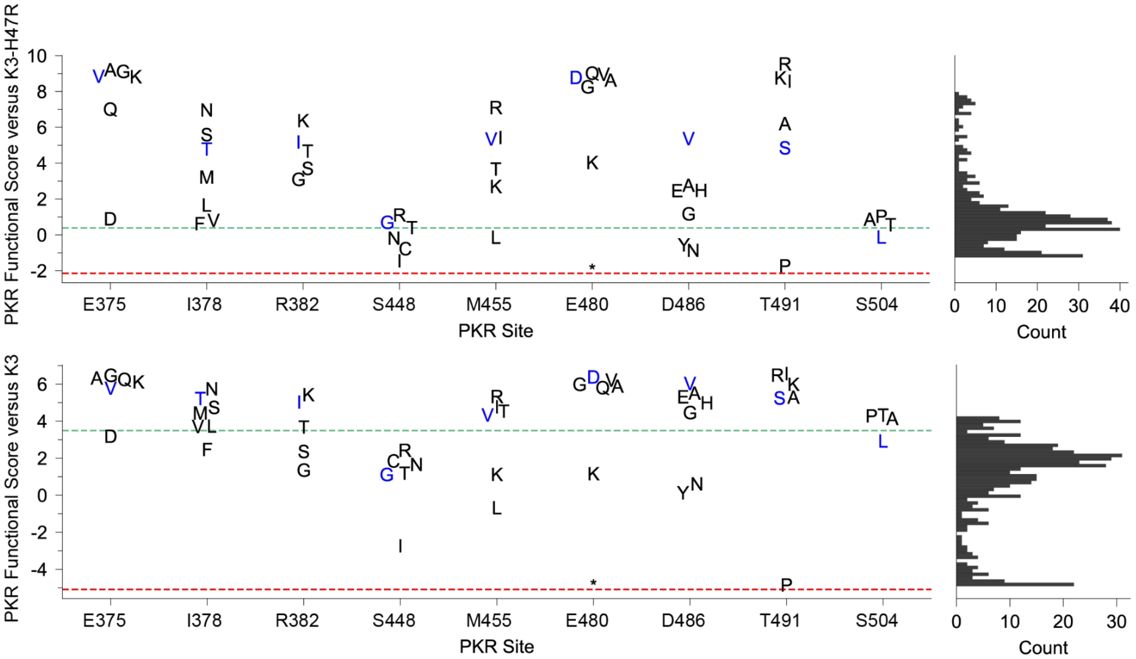

Supplemental Figure 6. Additional variants at the sites of previously identified K3-H47R-resistant variants often also conferred resistance to K3. Stripplots of PKR functional scores of variants paired with K3-H47R (top) and K3-WT (bottom). Each plot highlights PKR variants made at sites where an improved PKR variant was previously identified (28). Blue markers denote previously identified variants; black markers are additional variants made at the same site. Green and red dashed lines represent mean scores for WT PKR and nonsense variants, respectively. Histograms (right) show PKR functional scores versus K3-H47R (top) and K3 (bottom) for all variants. * = stop codon.

bioRxiv preprint doi: https://doi.org/10.1101/2024.05.29.596416; this version posted June 5, 2024. The copyright holder for this preprint (which was not certified by peer review) is the author/funder. This article is a US Government work. It is not subject to copyright under 17 USC 105 and is also made available for use under a CC0 license.

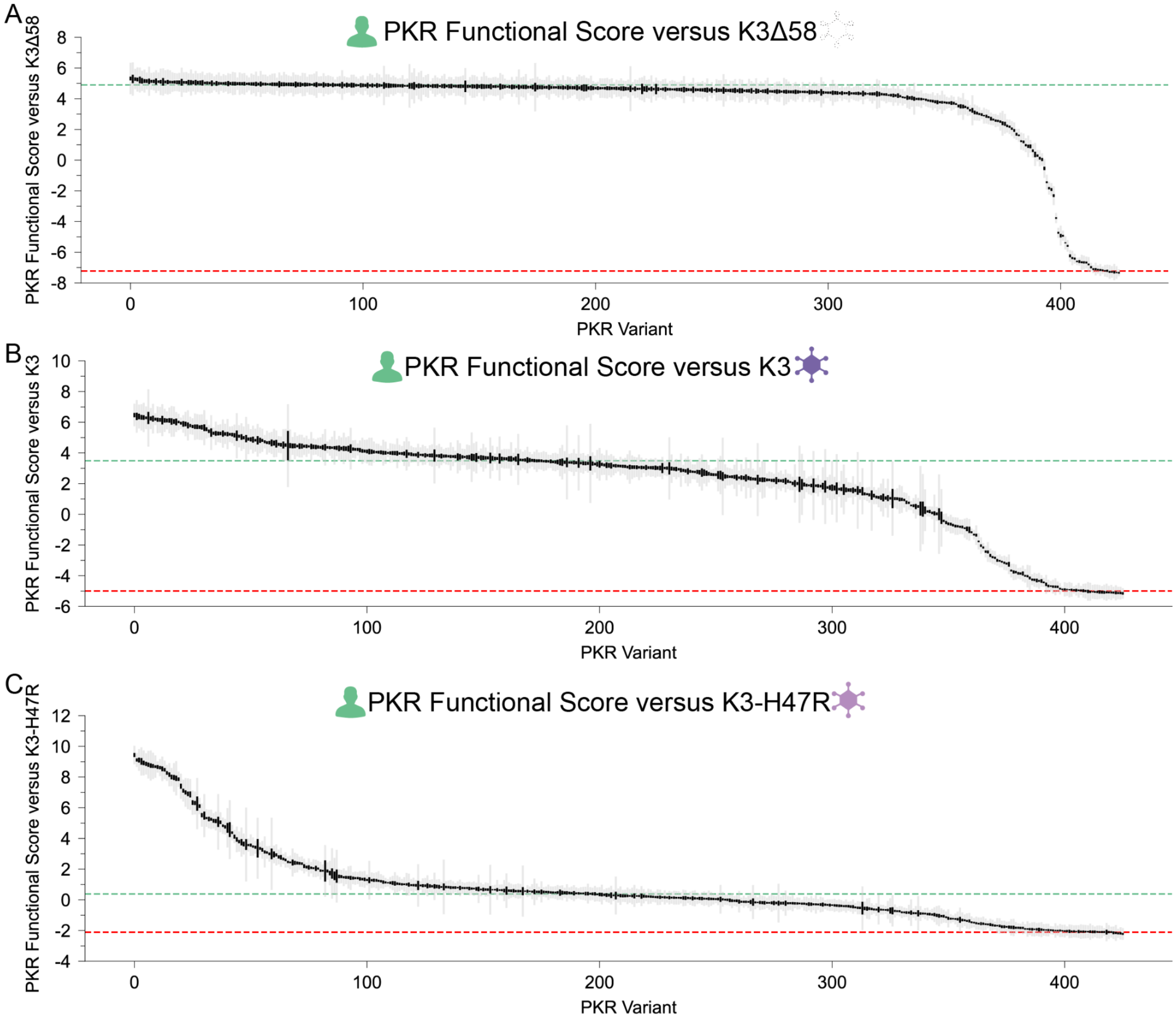

Supplemental Figure 7. Genetic functional resilience of PKR. PKR functional scores versus K3Δ58 (A), K3 (B), and K3-H47R (C), with each vertical line representing a PKR variant. Variants are sorted by functional score from high (left) to low (right), gray and black lines indicate the standard deviation and standard error, respectively in PKR functional scores across barcodes associated with the given variant.

bioRxiv preprint doi: https://doi.org/10.1101/2024.05.29.596416; this version posted June 5, 2024. The copyright holder for this preprint (which was not certified by peer review) is the author/funder. This article is a US Government work. It is not subject to copyright under 17 USC 105 and is also made available for use under a CC0 license.

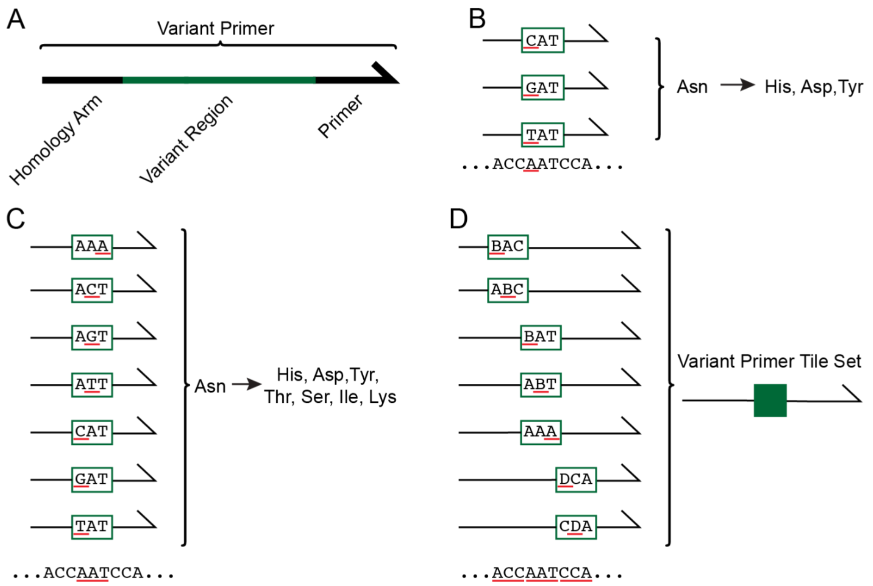

Supplemental Figure 8. Systematic generation of PKR variants using mixed-base primer tile sets. (A) Each variant primer is composed of a homology arm, variant region, and priming region. (B) Nonsynonymous SNP-accessible variants are generated by altering the codon in the variant region of the primer. This example depicts the codon “AAT” encoding Asn. The first nucleotide in the codon, “A”, is underlined in red, with three codons above having changes to “C”, “G”, and “T” underlined in red, which generate the nonsynonymous variants His, Asp, and Tyr. (C) Variant primers were designed across all three nucleotides in each codon, as underlined in red. (D) Variant primer tile sets, represented in dark green, were made by pooling variant primers that modify adjacent codons. Primers included in a given variant primer tile sets have differing variant regions but share homology arms and priming region sequences.

bioRxiv preprint doi: https://doi.org/10.1101/2024.05.29.596416; this version posted June 5, 2024. The copyright holder for this preprint (which was not certified by peer review) is the author/funder. This article is a US Government work. It is not subject to copyright under 17 USC 105 and is also made available for use under a CC0 license.

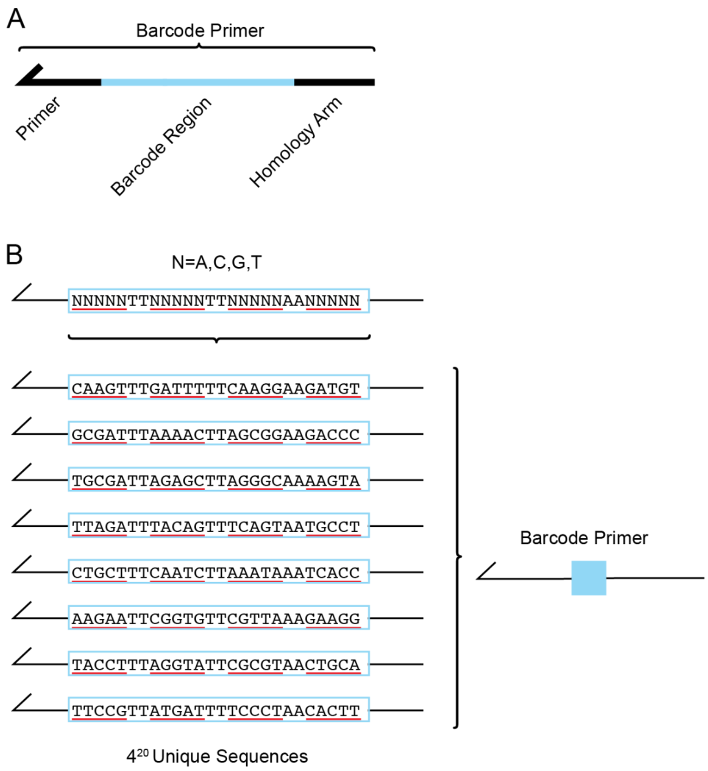

Supplemental Figure 9. Multiple unique barcode sequences were attached to each PKR variant. (A) The barcode primer is composed of a homology arm, barcode region, and priming region. The barcode primer is used as the reverse primer in the variant-generating PCR reactions to attach a unique nucleotide sequence after the PKR variant sequence. (B) The barcode region of the primer is composed of 20 "N", representing an equal mix of the nucleotides "A", "C", "G", and "T". A barcode primer with 20 random nucleotides can take on 4^{20} unique nucleotide sequences. Dinucleotide sequences "TT" and "AA" are interspersed throughout the barcode region to avoid making unintended restriction enzyme cut sites.

bioRxiv preprint doi: https://doi.org/10.1101/2024.05.29.596416; this version posted June 5, 2024. The copyright holder for this preprint (which was not certified by peer review) is the author/funder. This article is a US Government work. It is not subject to copyright under 17 USC 105 and is also made available for use under a CC0 license.

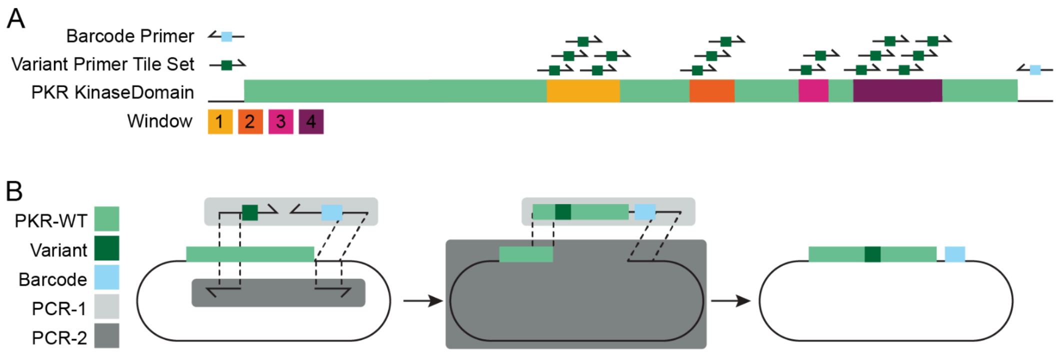

Supplemental Figure 10. Assembly of PKR variant library using variant tile sets and barcode primers. (A) 15 variant primer tile sets were designed to generate variants across four windows of interest in PKR. The full-length PKR sequence is denoted in green, with Windows 1-4 overlaid in yellow, orange, magenta, and burgundy, respectively. Variant primer tile sets were used to generate 426 PKR missense variants, for a total of 15 PCR-1 reactions. (B) Two separate PCR reactions were used to generate complementary insert and vector fragments. PCR-1 primers (light gray box) include a single variant primer tile set (dark green, see Supplemental Figure 8) and a single doped barcode primer (light blue, see Supplemental Figure 9) that amplified from WT PKR (green) and made the PCR-1 insert fragment containing a select nonsynonymous variants (dark green) and a unique barcodes (blue). PCR-2 primers included a single forward and reverse primer that amplified from WT PKR and made a larger vector fragment with 20-bp homology arms that complement the homology arms of the PCR-1 insert fragment. The two fragments were combined via Gibson Assembly to form a pool of complete vectors, each vector containing a single, nonsynonymous variant with a unique barcode.

bioRxiv preprint doi: https://doi.org/10.1101/2024.05.29.596416; this version posted June 5, 2024. The copyright holder for this preprint (which was not certified by peer review) is the author/funder. This article is a US Government work. It is not subject to copyright under 17 USC 105 and is also made available for use under a CC0 license.

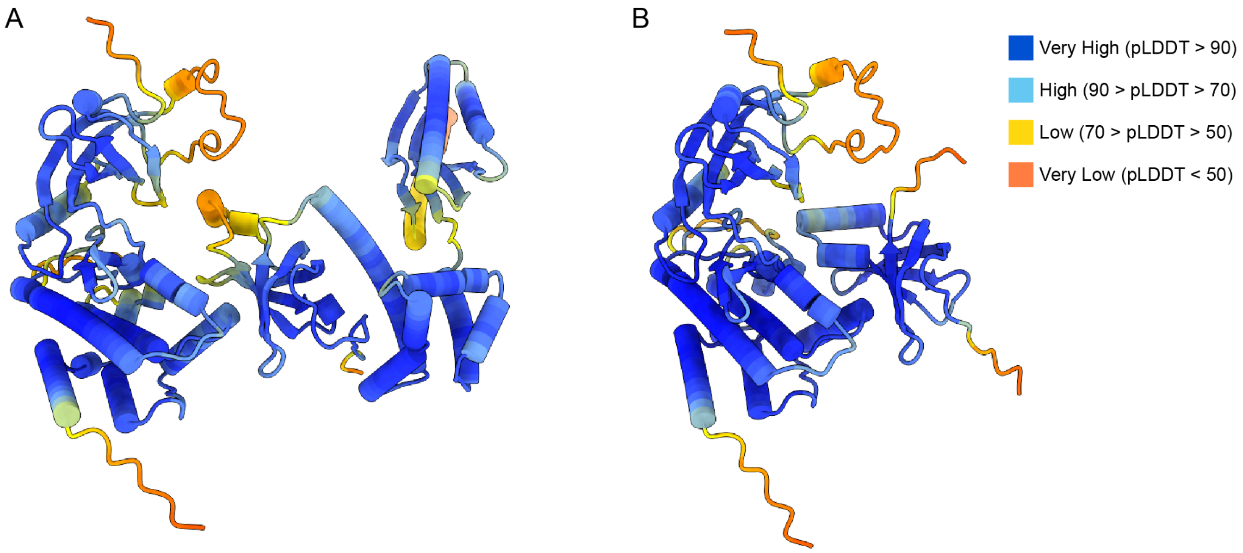

Supplemental Figure 11. Alphafold2 Multimer predictions used to identify PKR sites proximal to eIF2α and K3. Cartoon representations of AlphaFold2 Multimer predictions of PKR in complex with eIF2α (A) and K3 (B). Residues are colored AlphaFold2 pLDDT confidence scores per residue. In both panels, PKR is on the left and its binding partner is on the right, as in Figure 1A,B.

bioRxiv preprint doi: https://doi.org/10.1101/2024.05.29.596416; this version posted June 5, 2024. The copyright holder for this preprint (which was not certified by peer review) is the author/funder. This article is a US Government work. It is not subject to copyright under 17 USC 105 and is also made available for use under a CC0 license.

SUPPLEMENTAL DATA

Supplementary File 1 - 1_Oligo-Table.xlsx
Supplementary File 2 - 2_Variant-Primers.xlsx
Supplementary File 3 - 3_Plasmid-Table.xlsx
Supplementary File 4 - 4_PKR-Functional-Score-Table.csv
